# Percutaneous Suture Closure of Patent Foramen Ovale With Right Atrial Septal Pouch

**DOI:** 10.1016/j.jaccas.2026.109239

**Published:** 2026-07-09

**Authors:** Francesco Maria Animati, Gregory Angelo Sgueglia, Antonella De Santis, Fabrizio D'Errico, Maria Iamele, Tommasa Siragusa, Achille Gaspardone

**Affiliations:** a Department of Cardiovascular and Pulmonary Sciences, Catholic University of the Sacred Heart, Rome, Italy; b Division of Cardiology, Sant'Eugenio Hospital, Rome, Italy

**Keywords:** direct suture closure, patent foramen ovale, right atrial septal pouch, structural heart intervention, transesophageal echocardiography

## Abstract

**Background:**

Right atrial septal pouch is an uncommon septal variant that may predispose to thrombosis. When associated with patent foramen ovale (PFO), it may provide a substrate for paradoxical embolism.

**FIH/Early Reports Summary:**

A 52-year-old woman with recurrent cryptogenic transient ischemic attacks underwent transesophageal echocardiography showing PFO, a small cribriform secundum defect, and right atrial septal pouch with right-to-left shunt. She underwent fluoroscopy and echocardiography-guided percutaneous direct suture closure. Postprocedural imaging confirmed complete shunt abolition and pouch inversion/collapse, without complications. At 30 days, she remained asymptomatic, with no residual shunt or thrombus.

**Discussion:**

This case supports the feasibility of suture-mediated PFO closure in complex septal anatomy requiring both shunt closure and pouch exclusion.

**Novelty:**

This is, to our knowledge, the first reported direct suture closure of PFO associated with right atrial septal pouch.

**Take-Home Message:**

Direct suture closure may offer a device-free alternative in selected PFO patients with complex anatomy.

## Visual Summary

**Figure:**
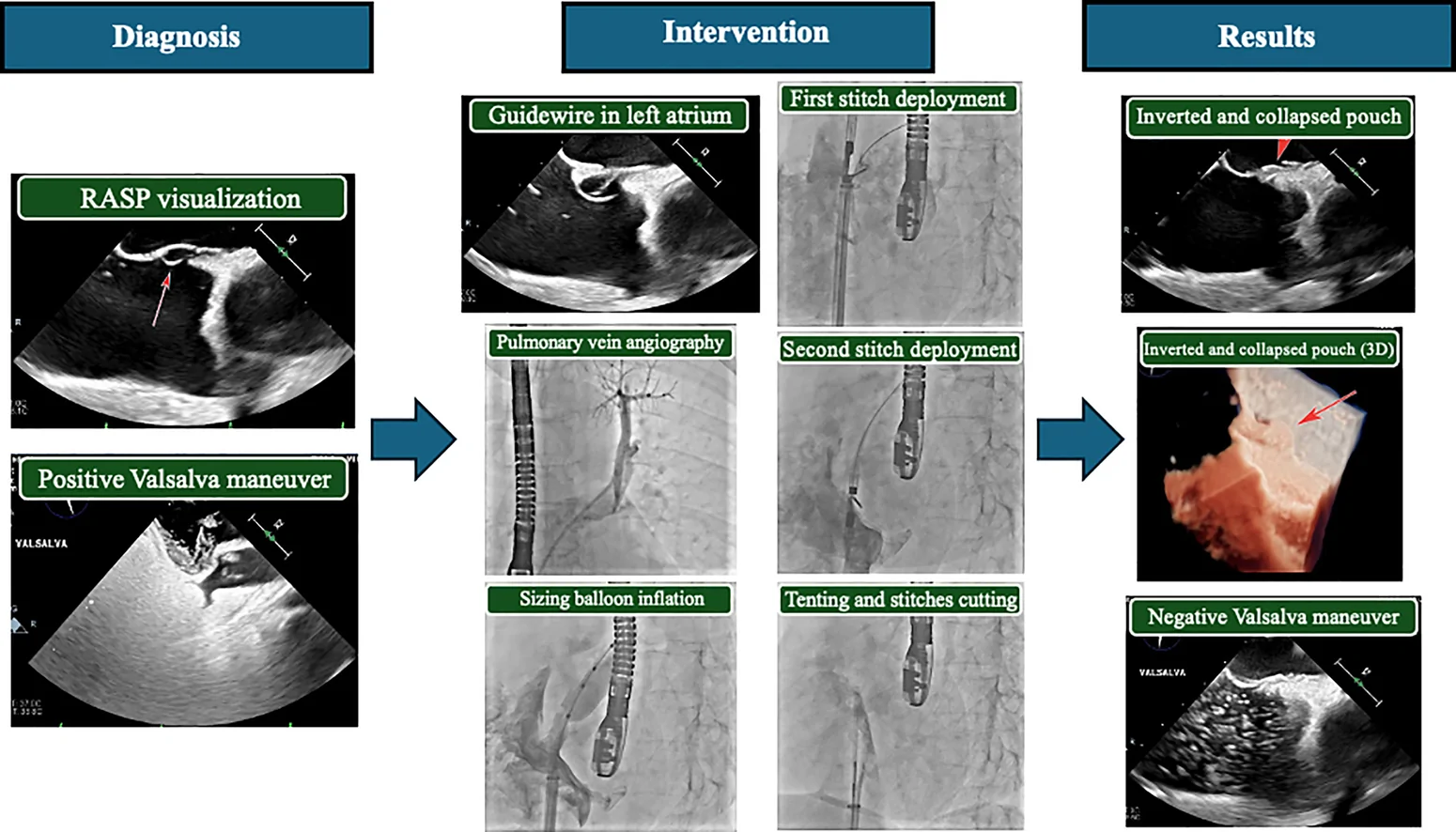

**Visual Summary:**
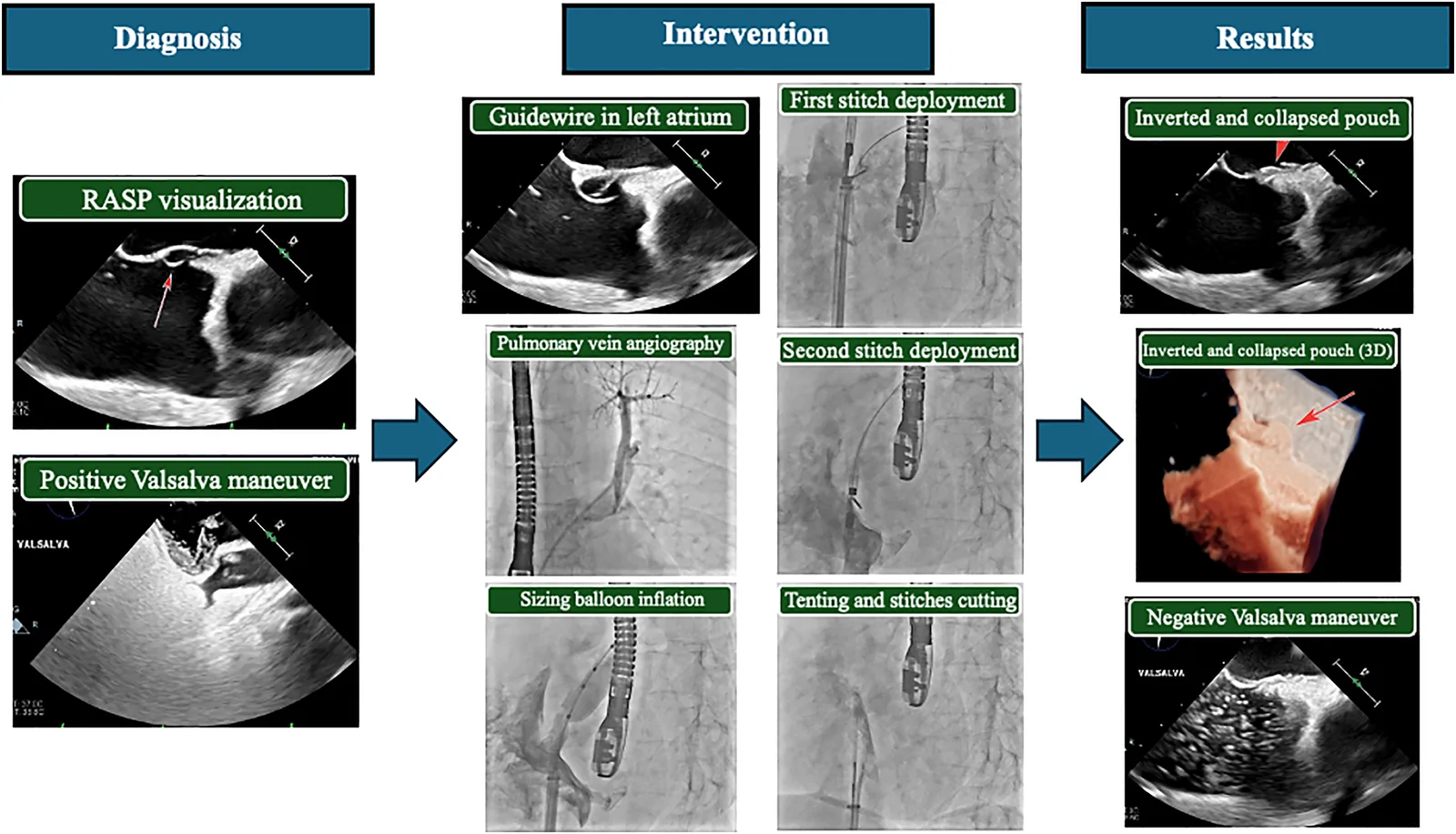
Stepwise Percutaneous Suture Closure of PFO With Right Atrial Septal Pouch Schematic overview of percutaneous direct suture closure of patent foramen ovale associated with right atrial septal pouch (RASP), illustrating the preprocedural anatomical assessment, stepwise suture-mediated closure of the septum secundum and septum primum, septal approximation, and final confirmation of effective closure. Both 2-dimensional and 3-dimensional (3D) transesophageal echocardiography demonstrate inversion and collapse of the pouch toward the left atrium after suture deployment, with complete abolition of the right-to-left shunt on agitated saline contrast testing during the Valsalva maneuver.

## Background

Atrial septal pouch, first described by Krishnan and Salazar^1^ in 2010, is a congenital variant arising from incomplete septal fusion, resulting in a pouch-like structure opening toward the left atrium, right atrium, or both, without an interatrial shunt. The right atrial septal pouch (RASP), the less frequent variant, has been associated with intracavitary thrombosis and pulmonary embolism.^2^

**Take-Home Messages**

• In patients with patent foramen ovale and right atrial septal pouch, successful treatment may require not only shunt closure but also functional exclusion of the pouch as a potential thromboembolic substrate.
• Percutaneous direct suture closure may provide a device-free strategy to achieve both shunt abolition and pouch exclusion, while preserving the interatrial septum and avoiding permanent intracardiac implantation.

We report a case of a patient with cryptogenic transient ischemic attack and concomitant patent foramen ovale (PFO) and RASP treated by percutaneous direct suture closure with NobleStitch EL suture delivery system (HeartStitch).

## Summary

A 52-year-old woman with no conventional cardiovascular risk factors and a history of recurrent cryptogenic transient ischemic attacks was referred for further evaluation.

**Figure 1:**
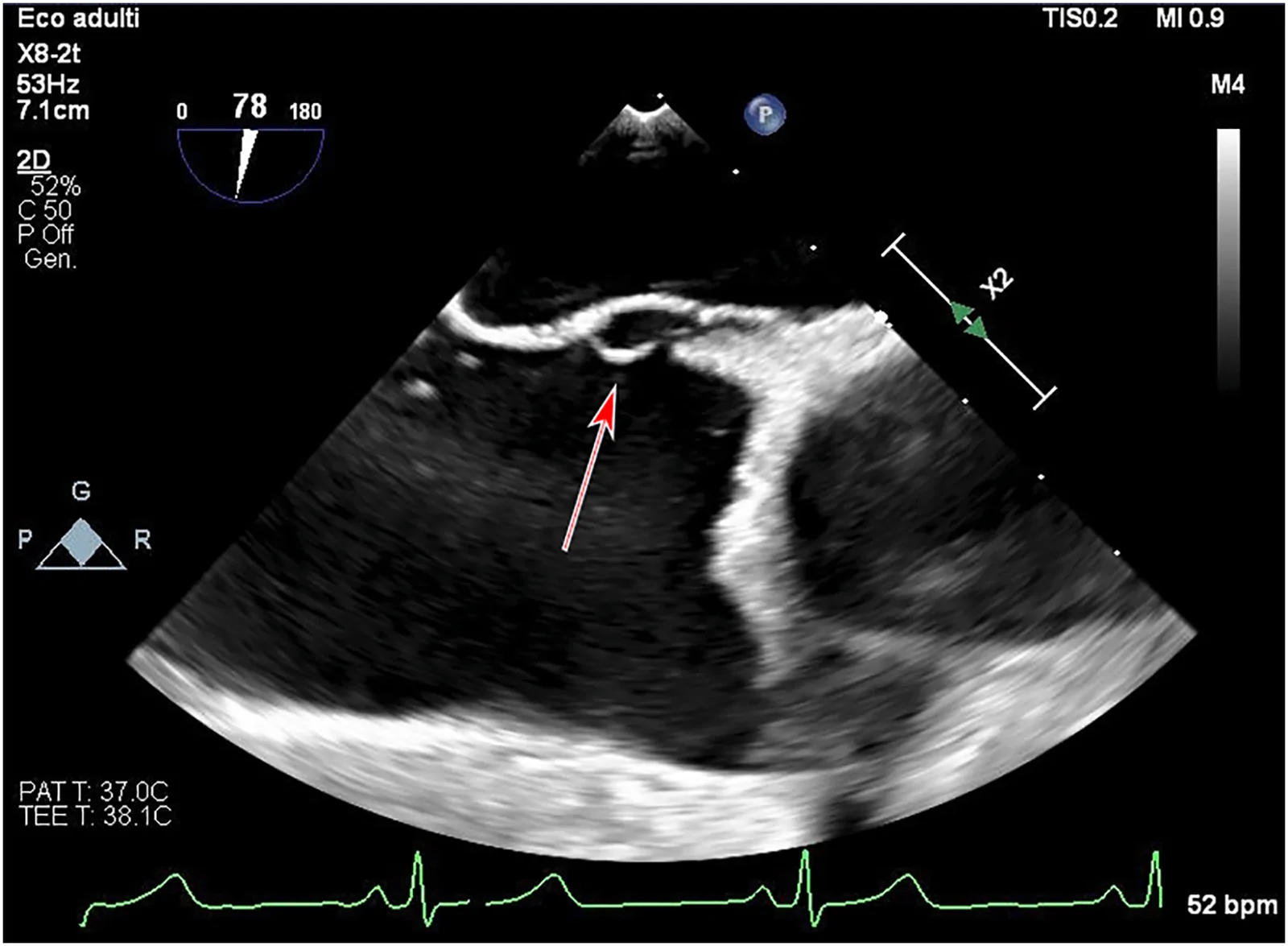
The Atrial Septal Pouch (Red Arrow) Can Be Seen Protruding into the Right Atrium

**Figure 2:**
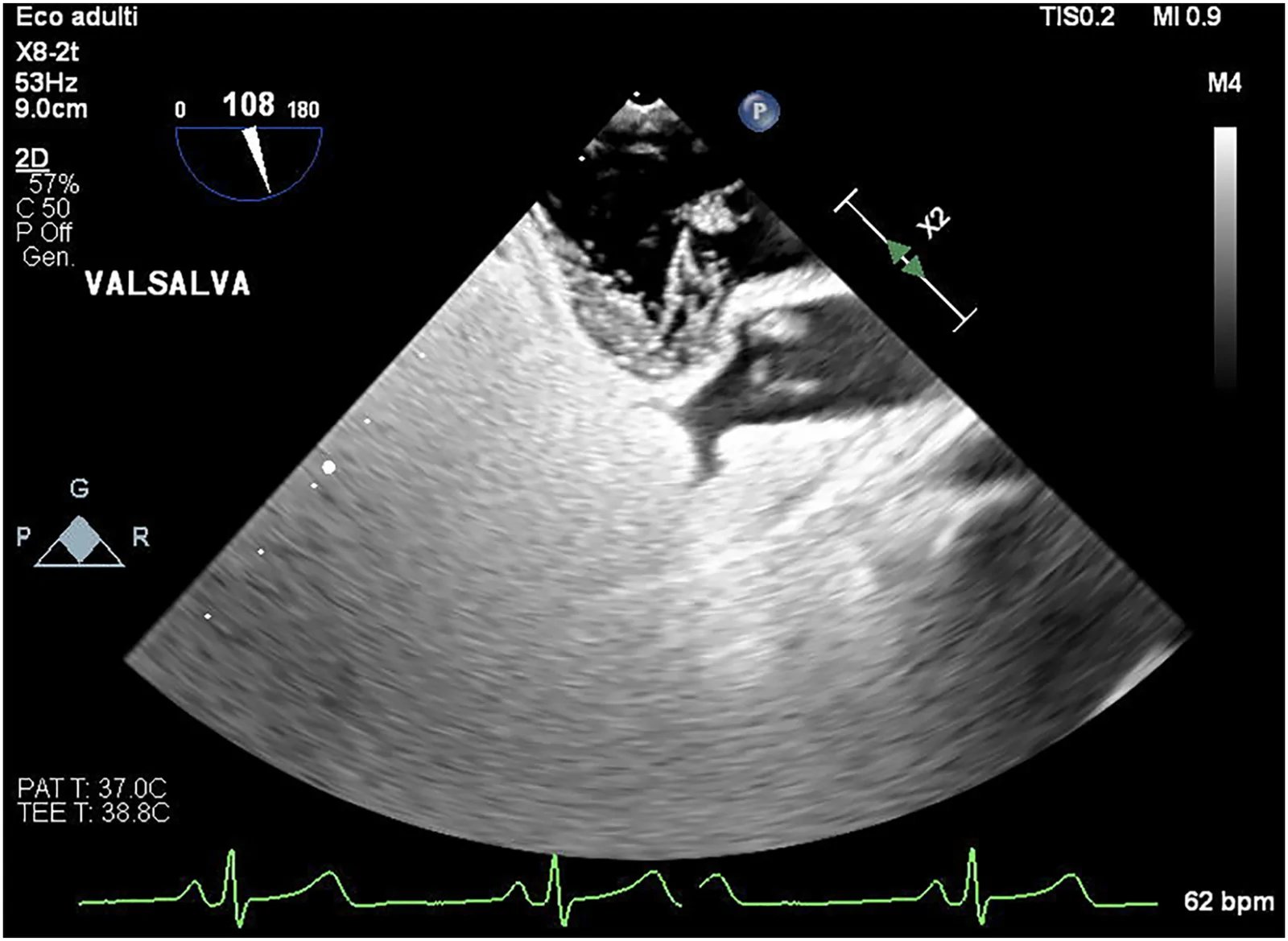
Agitated Saline Contrast Study During the Valsalva Maneuver Demonstrated Passage of Microbubbles From the Right to the Left Atrium Through the Patent Foramen Ovale, Consistent With a Moderate-to-Severe Right-to-Left Shunt

Transesophageal echocardiography revealed a PFO, a small additional cribriform defect consistent with a secundum atrial septal defect, a RASP (Figure 1). Agitated saline contrast study during the Valsalva maneuver demonstrated a moderate-to-severe right-to-left shunt (Figure 2). Based on these findings, also considering the preprocedural transesophageal echocardiographic assessment, which showed PFO anatomical features considered suitable for an attempted direct suture approach with a Length, Aneurysm, Severe Shunt, Opening (LASSO) score of 3,^3^ the patient was considered a candidate for percutaneous PFO closure with a device-free strategy using direct suture closure.

### Procedure

**Figure 3:**
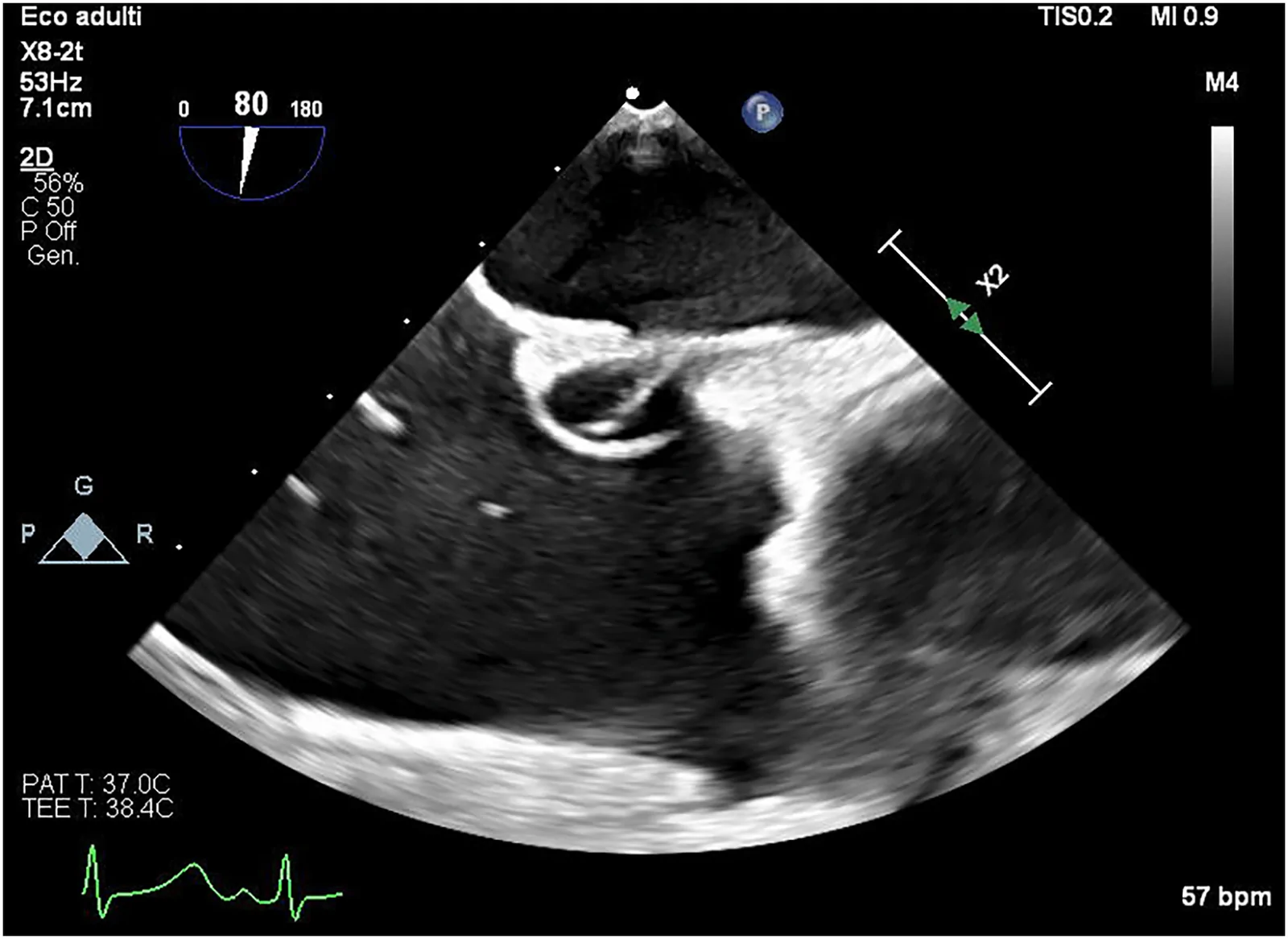
Successful Crossing of the Patent Foramen Ovale With a J-Wire Advanced into the Left Atrium

**Figure 4:**
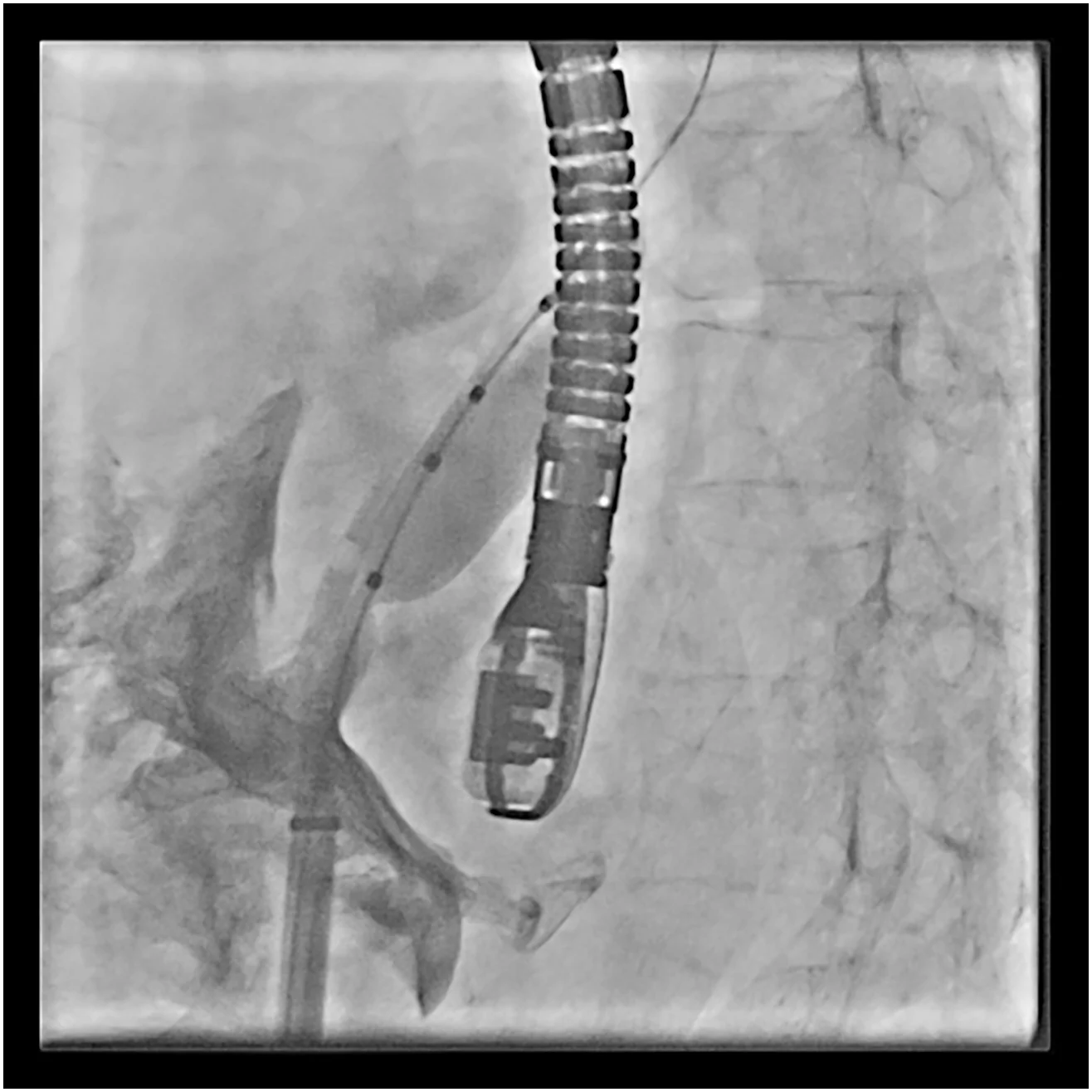
Inflation of the Sizing Balloon Within the PFO Tunnel, Allowing Measurement of a Tunnel Diameter of 28 mm

The procedure was performed under combined fluoroscopic and transesophageal echocardiographic guidance (Video 1). Right femoral venous access was obtained with preclosure using a suture-mediated vascular closure device, followed by insertion of a 6-F introducer. Intravenous heparin was administered to maintain an activated clotting time >250 seconds. The PFO was crossed with a J-wire advanced into the left atrium (Figure 3) through a multipurpose catheter. A 14-F sheathless catheter was then introduced, and a 25-mm sizing balloon was inflated to assess the PFO tunnel (Figure 4).

**Figure 5:**
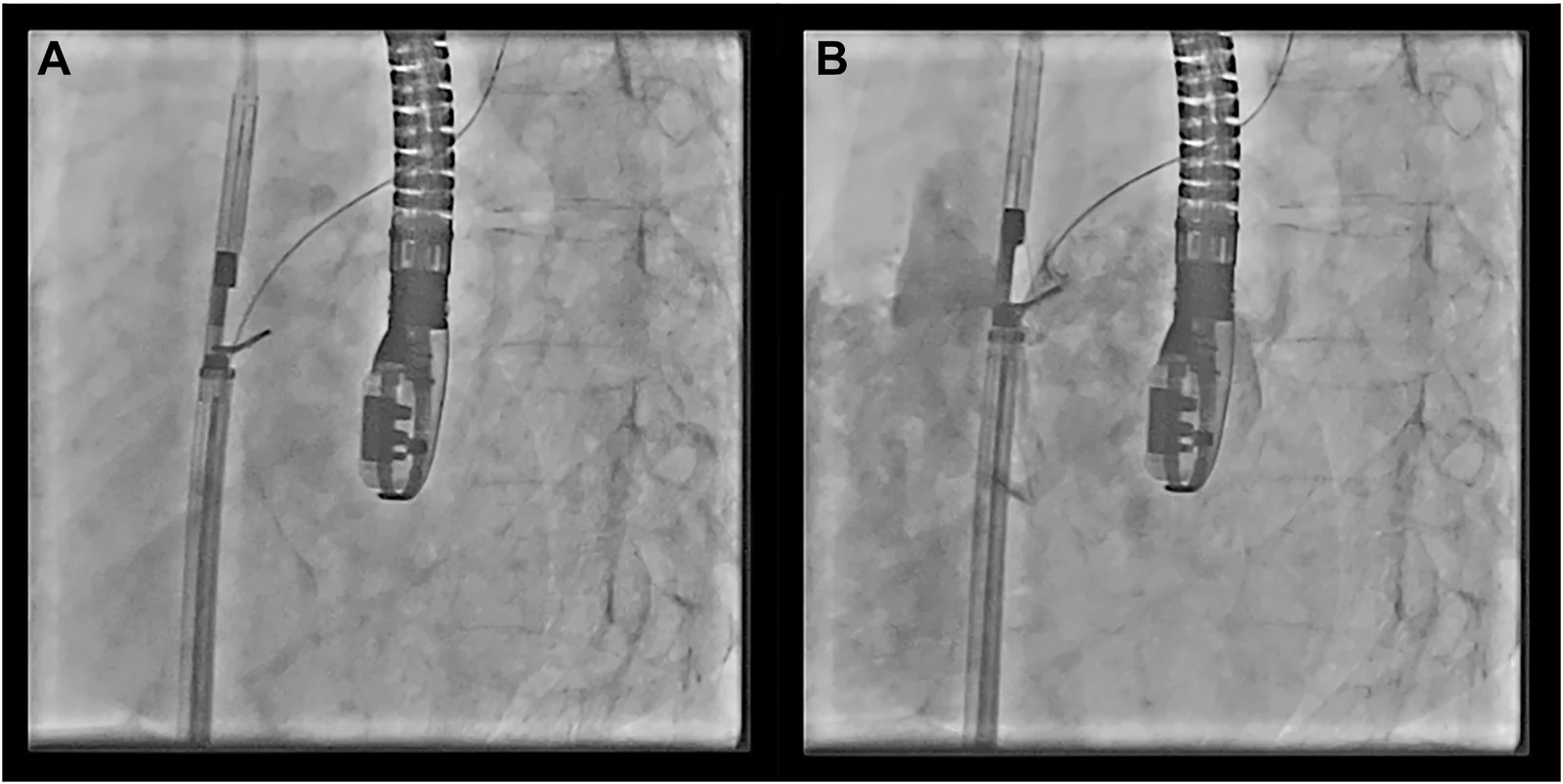
Fluoroscopy-Guided Septum Secundum Suture Deployment Fluoroscopic contrast guidance (A) followed by deployment of the suture through the septum secundum (B).

**Figure 6:**
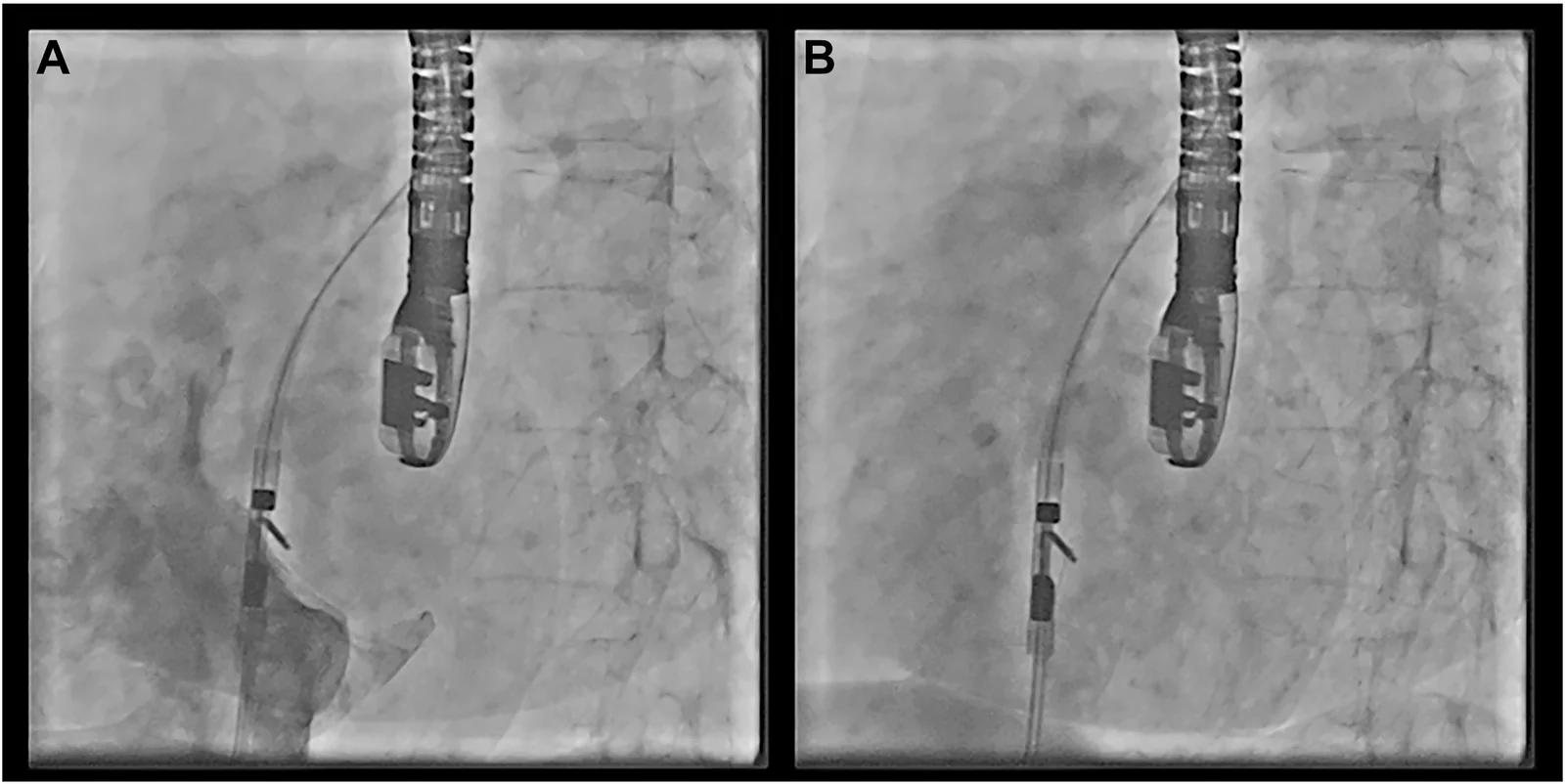
Fluoroscopy-Guided Septum Primum Suture Deployment Fluoroscopic contrast guidance (A) followed by deployment of the suture through the septum primum (B).

**Figure 7:**
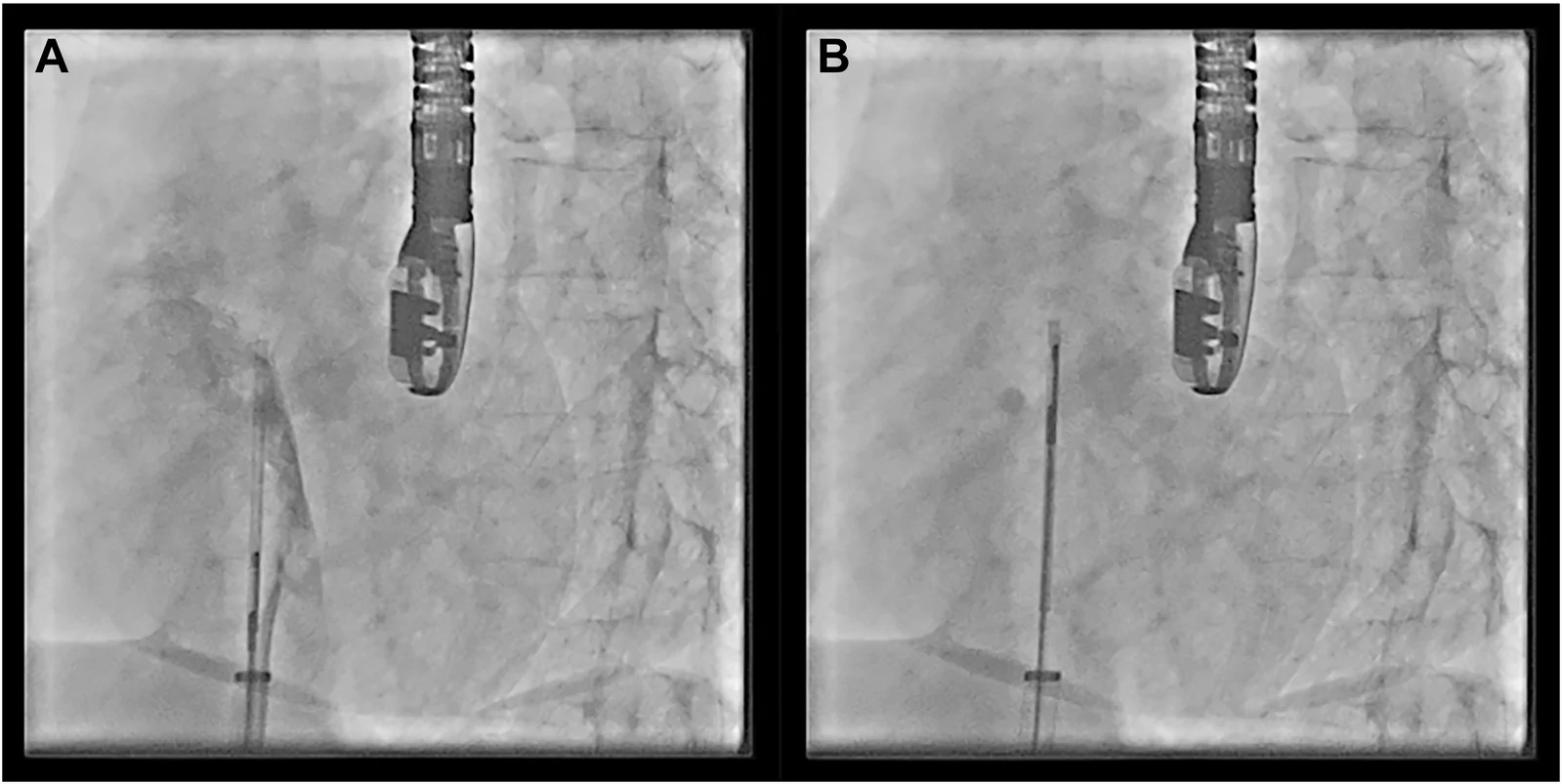
KwiKnot-Assisted Tenting and Locking Element Deployment Tenting performed with the KwiKnot catheter (A), followed by knot tightening and deployment of the locking element (B).

A 0.032-inch guidewire was positioned in the superior vena cava and a 0.018-inch guidewire across the PFO. The septum secundum was sutured using the dedicated secundum capture system (Figure 5), followed by suture placement through the septum primum using the dedicated primum capture system (Figure 6). A knot-delivery catheter was then used to perform septal approximation (tenting), confirming adequate tissue capture and septal apposition (Figure 7A). The suture was tightened and cut, and the locking element was deployed (Figure 7B).

### Postprocedural results

**Figure 8:**
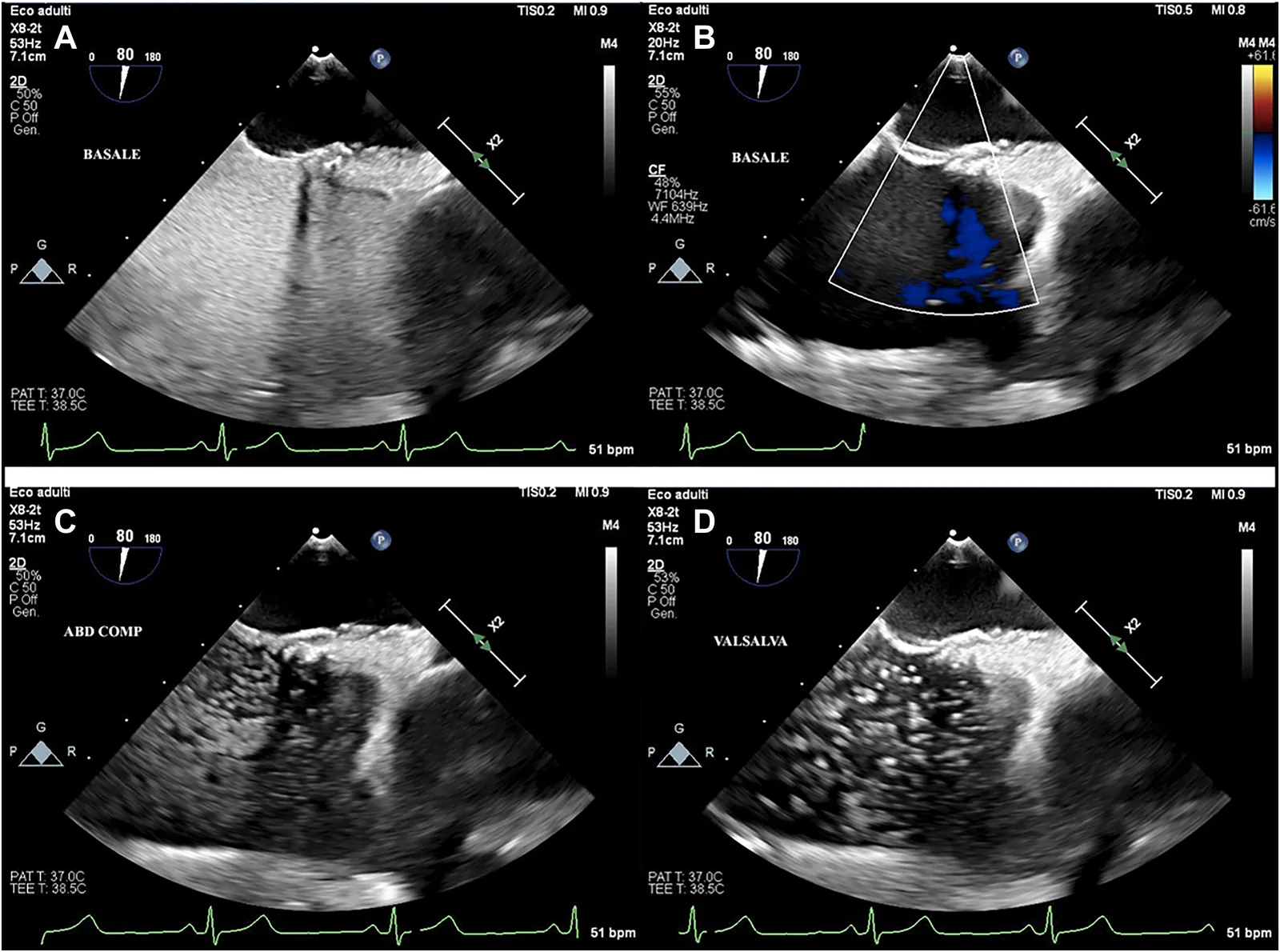
Post-Procedural Absence of Residual Right-to-Left Shunt The absence of agitated saline microbubble passage at rest (without color Doppler, A; with color Doppler, B), during abdominal compression (C), and during the Valsalva maneuver (D).

**Figure 9:**
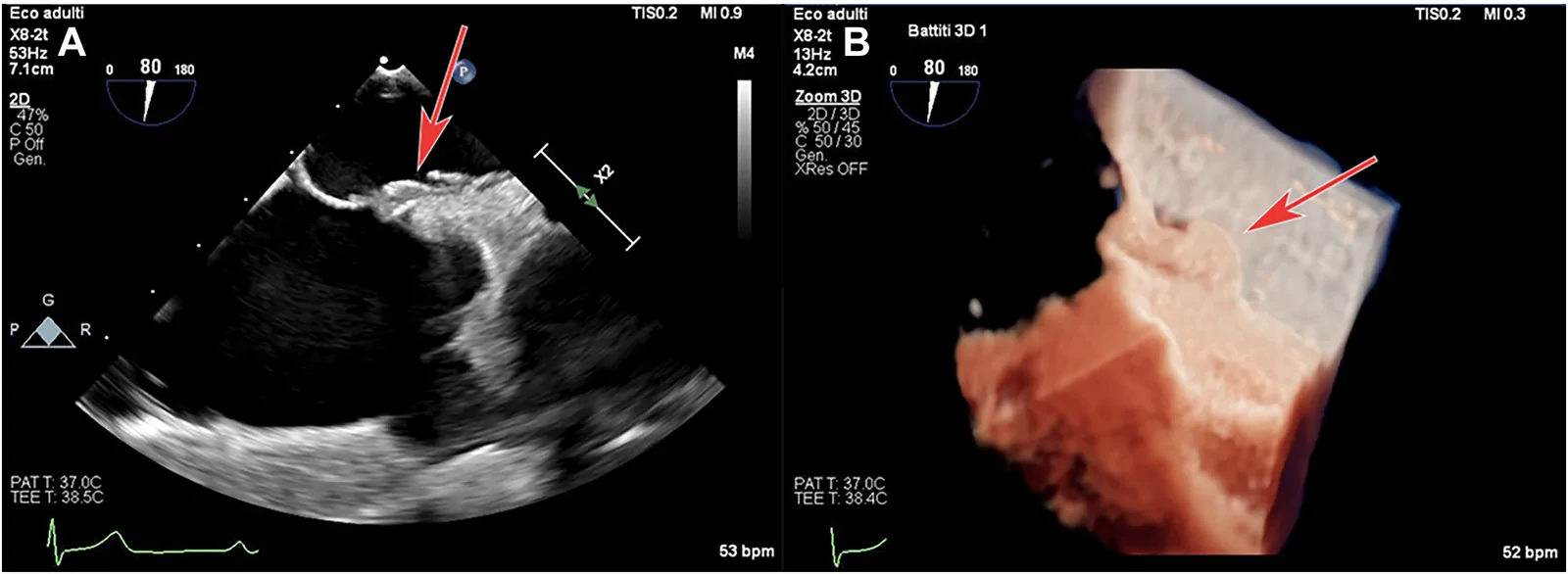
Post-Procedural Collapse and Left Atrial Inversion of the Septal Pouch The pouch (marked by red arrows), collapsed and inverted toward the left atrium on 2-dimensional transesophageal echocardiography (A) and 3-dimensional transesophageal echocardiography (B).

Transesophageal echocardiography demonstrated complete closure of the interatrial communication, absence of residual right-to-left shunt at rest, with abdominal compression, and during Valsalva maneuver (Figure 8), inversion and collapse of the pouch toward the left atrium (Figure 9). No procedure-related complications were observed, including atrial fibrillation or other atrial arrhythmias, bleeding events, or access-site complications.

At 30-day clinical follow-up, the patient was in good clinical condition and reported no recurrent cerebrovascular events. Follow-up transthoracic echocardiography with agitated saline contrast confirmed the absence of microbubble passage, consistent with persistent abolition of the right-to-left shunt. No thrombus formation was detected.

## Discussion

RASP is a rare anatomical variant, significantly less frequent than the left-sided form.^1^ Its structure—a blind-ended cavity communicating with the right atrium—may predispose to thrombus formation.^2^ In the absence of a PFO, embolization would be expected to occur in the pulmonary circulation. However, in the present case, the coexistence of PFO likely enabled paradoxical embolism, explaining the patient's cerebrovascular event. Although a previous report has described device-based closure in similar settings,^4^ this case represents, to our knowledge, the first successful use of a direct suture technique. The percutaneous direct suture with NobleStitch EL suture delivery system (HeartStitch) offers several potential advantages: absence of permanent intracardiac implant, reduced risk of device-related atrial fibrillation, avoidance of prolonged dual antiplatelet therapy, preservation of septal anatomy for future transseptal procedures, and elimination of device-related complications (erosion, thrombosis, nickel hypersensitivity).^5^ For these reasons, in patients with cryptogenic stroke attributable to PFO, it is important to gather more data to understand if suture-mediated closure may one day be considered as a first-line closure strategy. Suitability for this approach should be systematically assessed before considering device-based closure, mainly based on anatomical parameters included in the LASSO score, which may help identify patients in whom effective abolition of the right-to-left shunt can be reasonably expected. In addition to this, this strategy does not preclude bailout options, because in the event of an unsuccessful suture result or significant residual shunt, either placement of an additional stitch or conversion to conventional occluder device implantation remains feasible. Because RASP-associated PFO presents additional technical challenges—complex 3-dimensional anatomy, need to achieve both shunt closure and pouch exclusion, increased importance of imaging guidance, potential variability in septal tissue capture—this case demonstrates that direct suture closure is feasible even in complex anatomical settings.

## Novelty

To the best of our knowledge, this is the first reported experience of percutaneous direct suture closure of a PFO associated with a RASP. We propose that, in selected patients with complex septal anatomy, this device-free strategy may represent a feasible anatomical and functional approach to achieve both interatrial shunt abolition and pouch exclusion while avoiding permanent intracardiac device implantation.

## Future Directions

Direct suture closure may be considered a potential future standard of care for patients with complex PFO anatomies as well. Its device-free nature may be particularly advantageous when preservation of future transseptal access is desirable or when avoidance of dual antiplatelet therapy is preferred. Further studies are needed to confirm procedural reproducibility, long-term safety, and clinical efficacy across different PFO anatomical subsets.

## Conclusions

Direct suture closure of PFO associated with RASP is feasible and effective. This approach may represent a valuable alternative to device-based closure in selected patients with complex septal anatomy.

## Funding Support and Author Disclosures

Dr Gaspardone performs proctoring activities for Kardia Asahi Intecc. All other authors have reported that they have no relationships relevant to the contents of this paper to disclose.

## Appendix

Video 1 Step-by-Step Procedural Workflow of Percutaneous Direct Suture Closure of Patent Foramen Ovale, Illustrating the Entire Intervention From Initial Crossing of the Interatrial Communication and Anatomical Assessment to Septum Secundum and Septum Primum Suture Capture, Septal Approximation, Knot Deployment, and Final Confirmation of Effective Closure
